# Human renal adipose tissue from normal and tumor kidney: its influence on renal cell carcinoma

**DOI:** 10.18632/oncotarget.27157

**Published:** 2019-09-10

**Authors:** Flavia Alejandra Bruna, Leonardo Rafael Romeo, Fiorella Campo-Verde-Arbocco, David Contador, Silvina Gómez, Flavia Santiano, Corina Verónica Sasso, Leila Zyla, Constanza López-Fontana, Juan Carlos Calvo, Rubén Walter Carón, Virginia Pistone-Creydt

**Affiliations:** ^1^ Instituto de Medicina y Biología Experimental de Cuyo (IMBECU), Centro Científico y Tecnológico Mendoza, Consejo Nacional de Investigaciones Científicas y Técnicas (CONICET), Mendoza, Argentina; ^2^ Centro de Medicina Regenerativa, Facultad de Medicina, Clínica Alemana, Universidad del Desarrollo, Santiago, Chile; ^3^ Departamento de Urología y Transplante Renal, Hospital Español de Mendoza, Mendoza, Argentina; ^4^ Instituto de Biología y Medicina Experimental (IBYME), Consejo Nacional de Investigaciones Científicas y Técnicas (CONICET), Buenos Aires, Argentina; ^5^ Departamento de Química Biológica, Facultad de Ciencias Exactas y Naturales, Universidad de Buenos Aires, Buenos Aires, Argentina; ^6^ Universidad Nacional de Cuyo, Facultad de Ciencias Médicas, Departamento de Fisiología, Mendoza, Argentina; ^7^ Universidad Nacional de Cuyo, Facultad de Odontología, Mendoza, Argentina

**Keywords:** human renal adipose tissue, renal epithelial cells, cancer, epithelial-stromal interactions, migration

## Abstract

Tumor cells can interact with neighboring adipose tissue. We evaluated components present in human adipose explants from normal (hRAN) and kidney cancer (hRAT) tissue, and we evaluated the effects of conditioned media (CMs) from hRAN and hRAT on proliferation, adhesion and migration of tumor and non-tumor human renal epithelial cell lines. In addition, we evaluated the expression of AdipoR1, ObR, CD44, vimentin, pERK and pPI3K on cell lines incubated with CMs. hRAN were obtained from healthy operated donors, and hRAT from patients who underwent a nephrectomy. hRAT showed increased levels of versican, leptin and ObR; and decreased levels of perilipin, adiponectin and AdipoR1, compared to hRAN. Cell lines showed a significant decrease in cell adhesion and increase in cell migration after incubation with hRAT-CMs vs. hRAN- or control-CMs. Surprisingly, HK-2, 786-O and ACHN cells showed a significant decrease in cell migration after incubation with hRAN-CMs vs. control-CMs. No difference in proliferation of cell lines was found after 24 or 48 h of treatment with CMs. AdipoR1 in ACHN and Caki-1 cells decreased significantly after incubation with hRAT-CMs vs. hRAN-CMs and control-CMs. ObR and CD44 increased in tumor line cells, and vimentin increased in non-tumor cells, after incubation with hRAT-CMs vs. hRAN-CMs and control-CMs. We observed an increase in the expression of pERK and pPI3K in HK-2, 786-O and ACHN, incubated with hRAT-CMs. In conclusion, results showed that adipose microenvironment can regulate the behavior of tumor and non tumor human renal epithelial cells.

## INTRODUCTION

In addition to the epigenetic and genetic changes that occur in epithelial cells, in recent years it has been shown that tumor progression also depends on the bidirectional dialogue between tumor epithelial cells and surrounding stromal cells. Among the different types of cells that share microenvironment with renal epithelial cells, renal adipose tissue is one of the most abundant. Adipose tissue is a bioactive endocrine organ [1, 2], highly metabolic [3], that not only secretes soluble factors but also contributes significantly to the composition of the extracellular matrix (ECM). Adipose tissue has distinctive characteristics depending on its location [4]. For example, visceral adipose tissue presents anatomical, cellular and expression profiles different from subcutaneous adipose tissue [5]. Recent studies have shown that growth factors and cytokines secreted by adipose tissue have a significant impact on the progression of different diseases, including cancer [2, 6–12]. Renal cancer or renal cell carcinoma (RCC), is considered the fifth most frequent cancer type worldwide, and is related to a high mortality rate in both men and women [13]. RCC currently accounts for 90% of all new renal malignancies [14]. Established risk factors include tobacco smoking, body size, and history of hypertension and chronic kidney disease. Additionally, it has been suggested that additional underlying biological mechanisms might be responsible for disease occurrence. However, this hypothesis requires additional investigations [15]. It is known that the large amount of adipokines secreted by adipose tissue can act in an autocrine, paracrine and / or endocrine form and in this way control various cellular processes. Leptin and adiponectin are the most studied adipokines, since they are the most highly expressed by adipose tissue [16]. It has been postulated that leptin would have a pro-tumorigenic while adiponectin, an anti-tumorigenic function [17, 18]. Furthermore, it has been shown that leptin has pro-angiogenic effects while adiponectin can induce apoptosis of tumor cells [19–22]. An over-expression of leptin receptors (ObR) has been recently found in some cancer types [23]. In RCC patients, adiponectin levels are reduced and correlate inversely with the size of the tumor [24]. Versican may also interfere with tumor progression [25, 26]. Different studies have described that versican stimulates cell growth and inhibits cell adhesion [27], both processes related to tumor progression [28, 29]. The functional diversity of versican is dependent upon its concentration [30, 31]. In fact, it has been seen that the interaction between versican and CD44 affects the proliferation and motility of tumor cells [30, 32]. Versican is also cleaved by ADAMTS1 protease and its product of proteolysis is involved in migratory and vascularization processes [33]. Zi *et al.* [34] demonstrated that secreted factors from perineoplasm perinephric adipose tissue (PAT) might play a role in facilitating metastasis or perirenal fat invasion of clear-cell renal carcinoma (ccRCC), by mobilizing ccRCC cells away from primary tumor sites. We recently demonstrated that human adipose tissue from renal cell carcinoma near the tumor (hRATnT), regulates the behavior of tumor and non-tumor human renal epithelial cells differently than adipose tissue farther away from the tumor (hRATfT) [11]. Specifically, we observed that hRATnT-CMs differentially regulate the adhesion and migration of renal tumor and non-tumor epithelial cell lines, compared to hRATfT-CMs, without modifying their proliferation. In addition, we found that hRATnT secretes greater amounts of leptin and versican than hRATfT. Finally, we observed that human tumor and non-tumor renal epithelial cells incubated with hRATnT-CMs, decreased the expression of adiponectin type 2 receptor and modified the activation of PI3K and Akt, compared to the same cells incubated with hRATfT- or control-CMs [11]. In the present work, the microenvironment studied was human renal adipose tissue from: 1) patients with renal tumors (hRAT), and 2) healthy living kidney donors (hRAN). We identified soluble and non-soluble components present in the different fragments of adipose tissue (hRAN or hRAT), and their respective conditioned media (CMs), by qRT-PCR, Western blot and immunohistochemistry. In addition, we determined the effect of soluble factors released by these different adipose tissues on proliferation, adhesion and migration in different human renal cell lines (tumor and non-tumor); treated with the hRAN- or hRAT-CMs. Finally, we characterized factors that are modified in human renal cell lines, when incubated with hRAN- or hRAT-CMs. In particular, we evaluated: 1) changes in the expression of adiponectin, leptin receptors, CD44 as well as pERK and pPI3K as possible intracellular molecules that might be responsible for the different biological responses we studied; and 2) changes in the expression of vimentin, as a marker of the epithelial-mesenchymal transition, a characteristic process of epithelial cells when they acquire migratory capacity.

## RESULTS

### Versican and leptin gene expression was increased in hRAT compared to hRAN, while adiponectin gene expression was not modified

**Figure 1:**
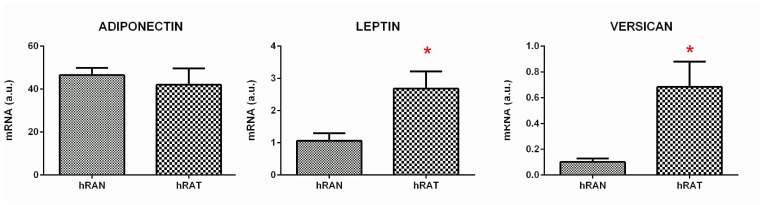
Relative fold expression of versican, adiponectin and leptin gene expression from hRAN and hRAT. The mRNA profiles of versican, adiponectin and leptin from different adipose tissue were analyzed by qRT-PCR and normalized by their relative ratio to GAPDH. Data are mean ± SEM. GAPDH, glyceraldehyde-3-phosphate dehydrogenase. ^*^ *p <* 0.05.

We measured gene expression (mRNA levels) of adiponectin, leptin and versican, in adipose tissue explants from normal (hRAN) and tumor (hRAT) kidney. Results showed an increase of versican and leptin mRNA level in hRAT compared to hRAN (Figure 1, *p <* 0.05). No significant differences were found in adiponectin mRNA expression (Figure 1).

### Perilipin 1 protein expression in hRAT-CMs showed decreased levels compared to hRAN-CMs, while adiponectin and ADAMTS 1 protein expression was not modified

Protein quantification (total amount) was performed in the conditioned media: hRAN-CMs: 2.12 ± 0.25 μg/μl (*n* = 12), and hRAT-CMs: 1.71 ± 0.29 μg/μl (*n* = 12) (*p <* 0.05).

**Figure 2:**
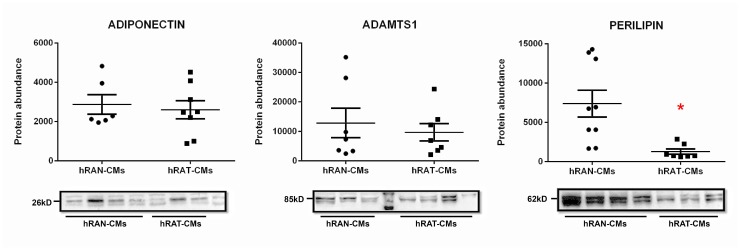
Adiponectin, ADAMTS1 and perilipin 1 in hRAN- and hRAT-CMs. Adiponectin, ADAMTS1 and perilipin 1 expression was evaluated by Western blot. Images were analyzed by densitometry. Horizontal bars represent the geometric mean of each data set. Vertical bars indicate SEM. ^*^ *p* < 0.05 hRAN-CMs *vs*. hRAT-CMs.

We evaluated the expression of perilipin1, adiponectin and ADAMTS1 1 in hRAT- and hRAN-CMs. Our results indicated a decreased expression of perilipin 1 in hRAT-CMs compared to hRAN-CMs (Figure 2, *p <* 0.01). This result could indicate that adipocytes from a tumor’s microenvironment have a less differentiated state than adipocytes from a normal microenvironment. No significant differences were found in adiponectin and ADAMTS1 (Figure 2).

### ObR expression was increased while AdipoR1, adiponectin and perilipin 1 protein expression was decreased in hRAT adipocytes compared to hRAN

**Figure 3:**
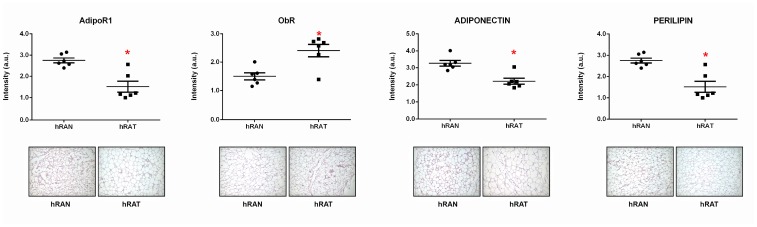
ObR, AdipoR1, adiponectin and perilipin 1 expression in the different adipose tissues. ObR, AdipoR1, adiponectin and perilipin 1 expression was evaluated by immunohistochemistry in serial cuts of hRAN and hRAT. DAB staining quantification in the three tissue types was performed with Image J software (NIH). Histograms show mean ± SEM of six independent experiments. (a. u.: arbitrary units). ^*^ *p* < 0.01 hRAN *vs.* hRAT. Representative photographs of hRAN- and hRAT-staining. Magnification: ×100.

In order to measure ObR, AdipoR1, adiponectin and perilipin 1 levels and localization in both tissue samples, we performed immunohistochemistry assays on adipose tissue explants from normal (hRAN) and tumor (hRAT) kidney. We found increased levels of ObR expression (Figure 3, *p <* 0.01) and a decrease in AdipoR1, adiponectin and perilipin 1 expression (Figure 3, *p <* 0.01), in hRAT adipocytes compared to hRAN adipocytes.

### Proliferation of 786-O, ACHN, Caki-1 (tumor) and HK-2 (non-tumor) cells was not modified by hRAN- or hRAT-CMs

**Figure 4:**
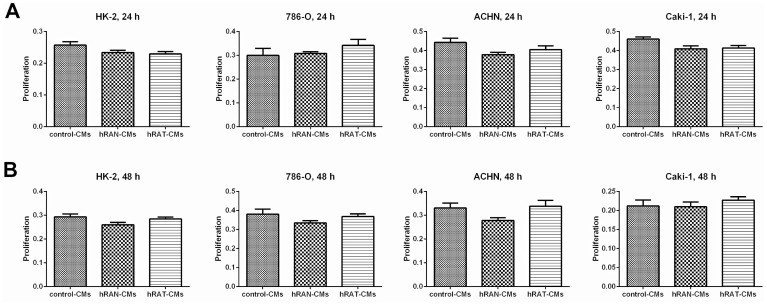
Figure 4: Effect of CMs from hRAN and hRAT on proliferation of HK-2, 786-O, ACHN and Caki-1 cell lines. HK-2, 786-O, ACHN and Caki-1 cell lines were incubated with hRAN- (*n *= 13), hRAT- (*n *= 14) or control-CMs for 24 (**A**) or 48 h (**B**). Proliferation was measured by MTT assays. Data are shown as the mean ± SEM (*n* = 4–5 experiments by triplicate).

In order to identify proliferation and cellular viability, an MTT assay and cell counting with Tripan blue were performed, finding in both cases consistent results. After incubating for 24 h or 48 h, proliferation was not modified in any of the cell lines studied, independently of the treatment performed (hRAN-, hRAT- or control-CMs) (Figure 4).

### Cellular adhesion of 786-O, ACHN, Caki-1 and HK-2 cells lines treated with hRAT-CMs was decreased. Adhesion of HK-2 (non-tumor) cells was increased after incubation with hRAN-CMs

**Figure 5:**
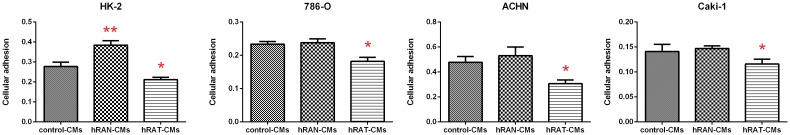
Effect of CMs from hRAN and hRAT on HK-2, 786-O, ACHN and Caki-1 cell lines attachment. HK-2, 786-O, ACHN and Caki-1 cell lines were plated at a density of 5 × 10^4^ cells/well in wells preincubated ON with hRAN- (*n* = 10–13), hRAT- (*n* = 10–13) or control-CMs and adherent cells were quantified by MTT. Data are shown as the mean ± SEM (n = 3 experiments by triplicate). ^*^ *p* < 0.05 hRAT-CMs *vs.* hRAN-CMs and control-CMs; ^**^ *p* < 0.05 hRAN-CMs *vs.* control-CMs.

786-O, ACHN, Caki-1 and HK-2 cells were seeded in plates previously exposed to different CMs. hRAT-CMs significantly reduced the adhesion of all cell lines compared to hRAN-CMs and control-CMs (Figure 5, *p <* 0.05). Surprisingly, HK-2 cells showed a significant increase in cell adhesion (*p <* 0.05) after incubation with hRAN-CMs *vs.* control-CMs.

### Migration of 786-O, ACHN, Caki-1 (tumor), and HK-2 (non-tumor) cells increased after incubation with hRAT-CMs, while the migration of 786-O, ACHN and HK-2 cells was decreased post incubation with hRAN-CMs

**Figure 6:**
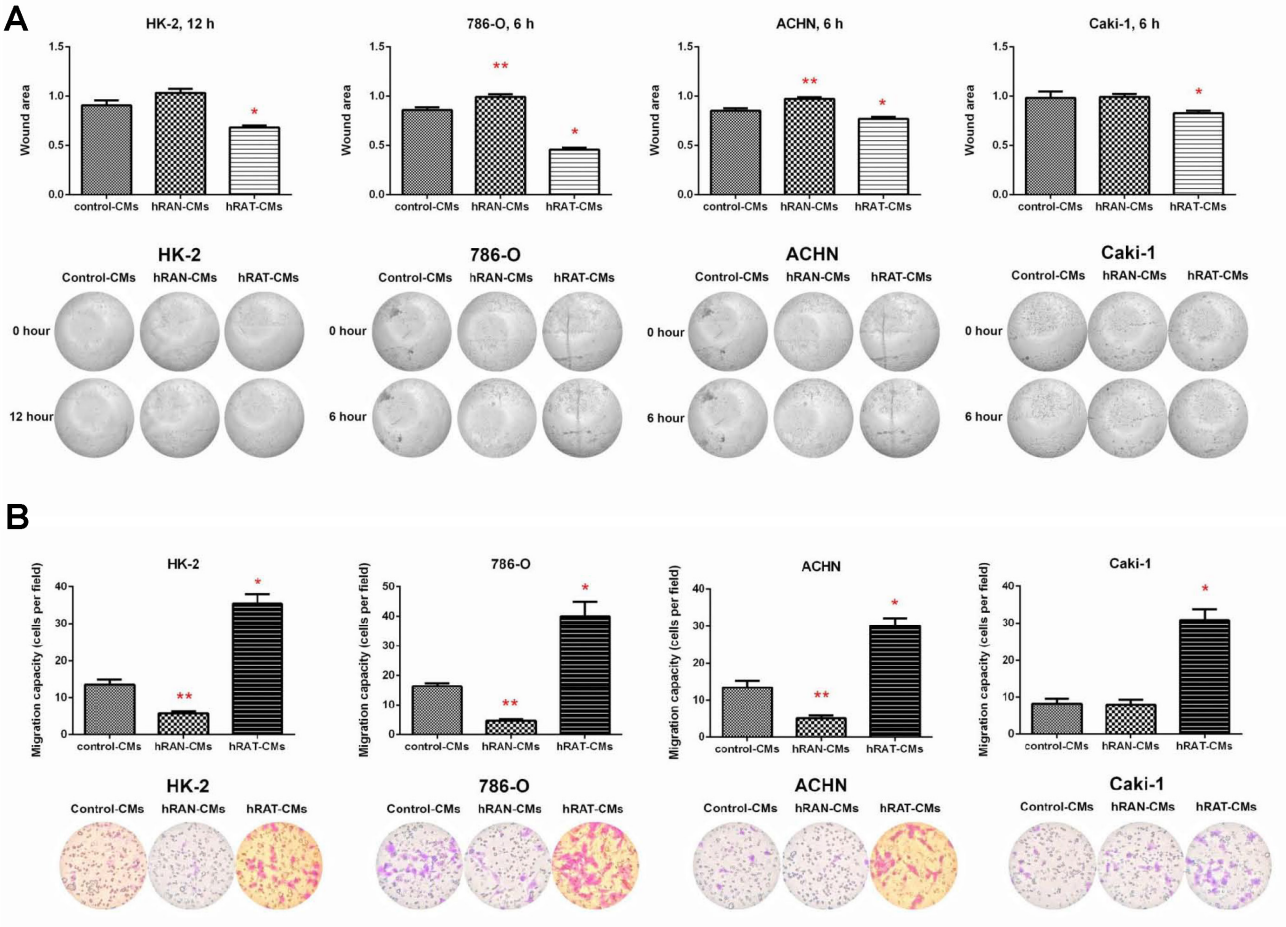
Effect of CMs from hRAN and hRAT on migration of HK-2, 786-O, ACHN and Caki-1 cell lines. Wound healing assay (**A**) HK-2, 786-O, ACHN and Caki-1 cell lines were grown with hRAN- (*n* = 10–13), hRAT- (*n* = 10–14) or control-CMs by additional 24 hs. After that, cells were wounded, washed twice with PBS and hRAN- (*n* = 13), hRAT- (*n* = 14) or control-CMs were added. Images were captured at the wound instant (0 h), after 6 and 12 hs. Representative light microscopic images (4×) are shown. HK-2, 786-O, ACHN and Caki-1 cell lines were incubated with hRAN-, hRAT- or control-CMs. The histogram shows the ratio of 12 hs/0 hs (HK-2) or 6 hs/0 hs (786-O, ACHN and Caki-1) cutting area and is plotted as mean ± SEM (*n* = 4 experiments by duplicate). ^*^ *p* < 0.001 hRAT-CMs *vs.* hRAN-CMs and control-CMs; ^**^ *p* < 0.05 hRAN-CMs *vs.* control-CMs. Transmigration assay (**B**): HK-2, 786-O, ACHN and Caki-1 cells were incubated with hRAN- (*n* = 10), hRAT- (*n* = 10) or control-CMs allowed to migrate across the porous membrane for 24 hs. The membranes were viewed under 20× magnification and migrated cells were counted in 5 randomly chosen fields per membrane. Data are shown as the mean ± SEM (*n* = 3 experiments by duplicate). ^*^ *p* < 0.001 hRAT-CMs *vs.* hRAN-CMs and control-CMs; ^**^ *p* < 0.05 hRAN-CMs *vs.* control-CMs.

After incubating for 6 h, hRAT-CMs significantly increased migration of 786-O, ACHN and Caki-1 (*p <* 0.001). Similarly, after incubating for 12 h, hRAT-CMs significantly increased (*p <* 0.001) migration of HK-2, *vs.* the effect of hRAN-CMs and control-CMs (Figure 6A). Transwell migration assays showed a similar pattern: transmigration of all cell lines increased significantly when they were incubated with hRAT-CMs *vs.* hRAN-CMs and control-CMs (*p <* 0.001) (Figure 6B). Interestingly, HK-2, 786-O and ACHN cells showed a significant decrease in cell migration (*p <* 0.05) after incubation with hRAN-CMs *vs.* control-CMs (Figure 6).

### In tumor renal cancer cells incubated with hRAT-CMs, AdipoR1 expression was diminished, while ObR and CD44 expression was increased. In addition, hRAT-CMs increased the expression of vimentin in HK-2 cells

We evaluated possible changes in the expression of AdipoR1, ObR, CD44 receptors and vimentin, in the different cell lines incubated with the CMs (hRAT-, hRAN- or control-CMs).

**Figure 7:**
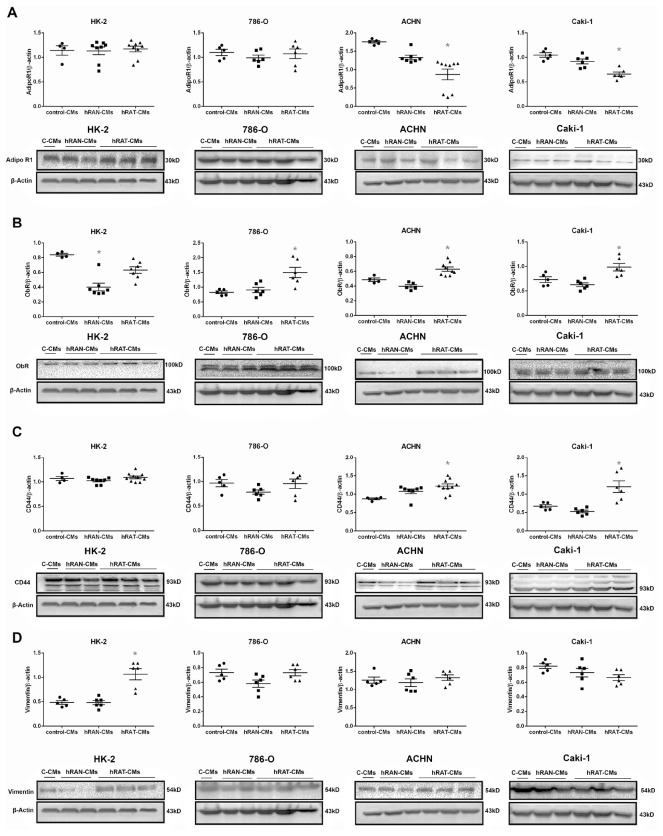
Effect of CMs from hRAN and hRAT on: AdipoR1 (**A**); ObR (**B**), CD44 (**C**) and vimentin (**D**) expression was evaluated in HK-2, 786-O, ACHN and Caki-1 cell lines. HK-2, 786-O, ACHN and Caki-1 cells were grown on 6 well plates, incubated for 24 hs with the different CMs and then lysed. Expression of the different proteins was measured by Western blot. β-actin was used as internal control. Images were analyzed by densitometry Horizontal bars represent the geometric mean of each data set. Vertical bars indicate SEM. ^*^ *p* < 0.001 cells incubated with hRAT-CMs vs. hRAN-CMs and control-CMs.

We observed a decrease in AdipoR1 expression in ACHN and Caki-1 (tumor cells) incubated with hRAT-CMs compared to hRAN- and control-CMs (*p <* 0.001) (Figure 7A). In addition, ObR expression in 786-O, ACHN and Caki-1 (tumor cells) was significantly higher when these cells were incubated with hRAT-CMs compared to hRAN- and control-CMs (*p <* 0.001) (Figure 7B). Additionally, CD44 expression in ACHN and Caki-1 cells was significantly higher when these cells were incubated with hRAT-CMs compared to hRAN- and control-CMs (*p <* 0.001) (Figure 7C). Finally, we observed an increase in vimentin expression in HK-2 (non-tumor cell) incubated with hRAT-CMs compared to hRAN- and control-CMs (*p <* 0.001) (Figure 7D).

### hRAT-CMs increased pERK expression in HK-2 and 786-O cells after 2 h of incubation, and increased pPI3K expression in HK-2, 786-O and ACHN cells after 24 h of incubation

**Figure 8:**
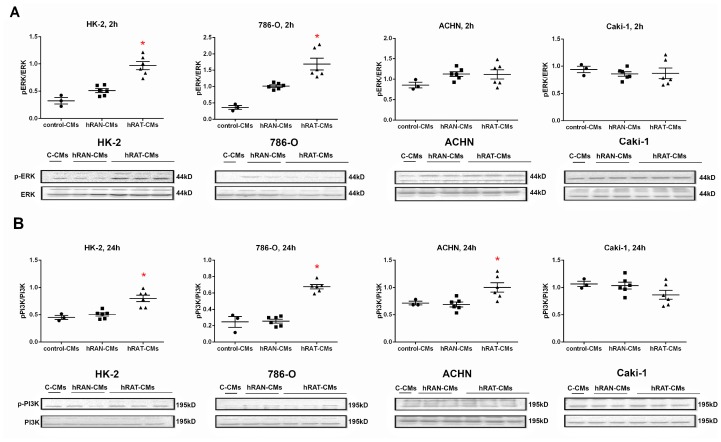
Effect of CMs from hRAN and hRAT on: pERK (**A**) and pPI3K (**B**) expression was evaluated in HK-2, 786-O, ACHN and Caki-1 cell lines. HK-2, 786-O, ACHN and Caki-1 cells were grown on 6 well plates, incubated for 24 hs with the different CMs and then lysed. Expression of the different proteins was measured by Western blot. ERK (A) and PI3K (B) were used as internal controls. Images were analyzed by densitometry Horizontal bars represent the geometric mean of each data set. Vertical bars indicate SEM. ^*^ *p* < 0.001 cells incubated with hRAT-CMs vs. hRAN-CMs and control-CMs.

In order to elucidate possible intracellular mechanisms involved in the observed biological effects (e. g. changes in cell adhesion and migration), we studied the expression of some intracellular proteins involved in different signaling pathways that activate gene transcription (for instance pERK and pPI3K). We found an increase in pERK expression in HK-2 and 786-O cells incubated for 2 h with hRAT-CMs compared to hRAN- and control-CMs (*p <* 0.001) (Figure 8A). Furthermore, pPI3K expression in HK-2, 786-O and ACHN cells increased significantly when these cells were incubated with hRAT-CMs compared to the value observed with hRAN- and control-CMs (Figure 8B, *p <* 0.001).

## DISCUSSION

In tumor development and maintenance of a cancerous phenotype (whether invasive or not), bidirectional communication between epithelial cells and the stromal environment is necessary. This communication can trigger a cancerous behavior as well as act in its maintenance or eventual involution to a non-tumor form. Our group has shown that periprostatic adipose tissue from prostate cancer patients could influence tumor behavior even at early stages [35]. Furthermore, we have worked with adipose tissue fragments from human breast tumors (hATT) and normal breast glands (hATN), from which we obtained the corresponding conditioned media (hATT-CMs and hATN-CMs, respectively) [9, 10, 12]. Based on these results, we could demonstrate the importance of epithelial cell microenvironment, in particular of human adipose tissue, in the regulation of growth and metastasic capacity of human breast epithelial cells. Thus, in the present study we aimed to evaluate the effect of human renal adipose tissue from normal and cancerous kidney on renal epithelial cells. Adipose tissue is the most prevalent tissue in the human body. It is commonly found in subcutaneous connective tissue and also surrounding various organs including the kidneys [24]. This tissue can produce and secrete a variety of cytokines [36, 37]. Among them is adiponectin, which has been linked to different diseases. Low serum levels of adiponectin have been related to obesity, insulin resistance, metabolic syndrome, atherosclerosis, and cancer [38, 39]. AdipoR1 (one of the adiponectin receptors), has been of interest in cancer research because of its anti-tumorigenic role described when interacting with its ligand [38, 40]. In agreement with the possibility that adipose tissue from a normal microenvironment has a protective role on the surrounding tissue, we found that hRAT have decreased levels of adiponectin and AdipoR1 compared to hRAN (Figure 3). On the other hand, leptin has an opposite function in comparison with adiponectin. In obese people, serum leptin levels are usually increased [3, 41]. Obesity is a risk factor for various diseases, including cancer [42]. Leptin is a hormone that regulates adipocyte size, as well as the development of certain tumor types. Its involvement in tumor development would seem to be related to its antiapoptotic, mitogenic and proangiogenic effects. In addition, leptin promotes murine renal cancer cell invasiveness through extracellular signal-regulated kinase signaling pathway, and guanosine-Rho triphosphate dependent pathways [43]. Therefore, leptin signaling could have a key role in renal cancer cell invasion. We found a significant increase in leptin and its ObR receptor in hRAT adipocytes with respect to hRAN adipocytes (Figures 1 and 3). In addition, in the present work, we found that adipocytes from tumor microenvironment express diminished levels of perilipin-1 (Figures 2 and 3). Perilipin-1 is found in the membrane surrounding lipid droplets and in the plasma membrane, and its expression is associated with mature adipocytes [44]. Some authors have observed that adipocytes exposed to factors secreted by tumor cells revert to a more undifferentiated state, and that the conditioned medium of these adipocytes promotes the migration of tumor epithelial cells [45]. Therefore, our results suggest that adipocytes from tumor microenvironment revert to a less differentiated state and that this probably implies a change in their functions. Likewise, we observed that versican is increased in hRAT compared to hRAN samples (Figure 1). Therefore, versican could be playing a role in fat cells. Several studies have shown that versican seems to be involved in diverse cell functions such as adhesion, migration and proliferation [32]. Interestingly, it has been described that during adipose differentiation processes, adipokines increase the production of proteoglycans (among them versican) in a 3T3-L1 fibroblastic murine cell line [46]. However, once cells reach a differentiated mature state, this production falls below the parental cell line level [47]. Thus, the increase of versican would appear to be related to the first stages of the differentiation process to adipocytes, and would be expressed by cells in intermediate stages of differentiation. Therefore, versican increase in hRAT adipocytes could be associated to the reversal of mature state adipocytes to a less differentiated state, in agreement with the observed decrease of perilipin-1 expression. The lower expression of versican by hRAN adipocytes thus correlates with a differentiated state of mature adipocytes. In summary, the identification of these specific proteins differentially expressed between hRAT and hRAN, would indicate that renal adipose tissue secretes factors that protect the organ against an aberrant cellular behavior, and that when a tumor appears, this protection is lost. In addition, we evaluated the importance of adipose tissue microenvironment in the regulation of the biological behavior of renal epithelial cells. To this end, we evaluated the effects of hRAT-CMs or hRAN-CMs on cell proliferation, adhesion, and migration of 786-O, ACHN, Caki-1 (tumor) and HK-2 (non-tumor) cell lines. All cell lines showed a significant decrease in cell adhesion (Figure 5), and increase in cell migration (Figure 6), after incubation with hRAT-CMs *vs.* hRAN- or control-CMs. These results suggest that adipokines secreted by hRAT (present in the CMs), are able to decrease cell adhesion, and stimulate cell migration of both tumor and non-tumor renal epithelial cells. This does not exclude though, that adipokines and other factors secreted by other tissues (near or farther away from the renal tumor), might also be promoting RCC development. Interestingly, HK-2, 786-O and ACHN cells showed a significant decrease in cell migration after incubation with hRAN-CMs *vs.* control-CMs (Figure 6). This result would indicate that adipose tissue surrounding a normal kidney (hRAN) has the ability to reverse, at least partially, the tumorigenic behavior of renal tumor epithelial cells. Therefore, it would be extremely important to identify those factors responsible for this observed effect, and to use them to limit renal tumor development. No differences on proliferation of cell lines were found after 24 or 48 h of treatment with CMs, in accordance with our previous results [11]. In order to elucidate the mechanisms underlying the biological effects observed in cell lines incubated with the different CMs, we assessed if there were changes in the expression of different proteins. We found a decrease in the expression of AdipoR1 in renal tumor cell lines ACHN and Caki-1 (from metastatic site) incubated with hRAT-CMs (Figure 7A). Additionally, an increase in the expression of ObR in 786-O, ACHN and Caki-1 (tumor cells) incubated with hRAT-CMs (Figure 7B) was found. Adiponectin usually has an anti-tumor behavior, and leptin increases cellular tumorigenicity. Therefore, ours results demonstrate that hRAT-CMs modify the phenotype of renal tumor cells towards a more tumorigenic behavior. Also, we found that hRAT-CMs increase CD44 expression in some tumor cell lines (Figure 7C). Numerous studies have shown that CD44, a membrane glycoprotein, is involved in processes of tumor cell migration and invasion [48, 49]. Moreover, CD44 is a marker, together with CD24, of tumor-initiating cells or tumor stem cells [50]. Considering this, our results could be indicating that hRAT-CMs increase the subpopulation of cells with high levels of CD44, and that, in this way, promote cellular invasion, resistance to treatments and malignancy. Regarding vimentin, this protein forms the intermediate filaments of the cytoskeleton and, in addition, is a marker of mesenchymal cells [51]. In particular, vimentin has received attention in recent years because it has been found overexpressed in different types of tumor cells, and this overexpression is associated with an increase in cell invasion and tumor growth [52]. Furthermore, vimentin is a marker of epithelial-mesenchymal transition, a process by which tumor epithelial cells acquire the ability to migrate, and is involved in cell invasion and metastasis [53]. We found that hRAT-CMs increase the expression of vimentin in a non-tumor cell line (Figure 7D), which indicates that factors present in the CMs from hRAT have the ability to revert the phenotype of non-tumor epithelial cells to a mesenchymal phenotype.

Finally, we evaluated changes in intracellular signaling pathways (ERK and PI3K) of normal and tumor cell lines incubated with the different conditioned media. Ours results showed an increase of pERK in HK-2 and 786-O cells incubated for 2 h with hRAT-CMs compared to hRAN- and control CMs (Figure 8A). In addition, we found a significant increase of pPI3K in HK-2, 786-O and ACHN cells incubated for 24 h with hRAT-CMs compared to hRAN- and control CMs (Figure 8B). These increases in pERK and pPI3K levels could at least partially explain the observed changes in migration and cell adhesion.

Based on the results presented, we postulate that renal peritumoral adipose tissue undergoes a process of adaptation to changes locally generated by the tumor. Thus, adipose tissue close to the tumor would favor tumor progression through factors secreted to its microenvironment (hRAT), unlike normal renal adipose tissue (hRAN). Specifically, we hypothesize that this hRAT, already modified with regard to hRAN, is capable of stimulating a protumorigenic behavior of renal epithelial cells. Likewise, the hRAN would be able to reverse the tumorigenic behavior of renal tumor epithelial cells. We acknowledge that the present study has some methodological limitations. Firstly, we measured only a few of all possible adipokines. We expect to be able to identify other relevant factors by means of proteomic analysis. Secondly, we have focused only in one of the several stromal cell types (adipocytes) that could be influencing tumor behavior. Nevertheless, despite possible limitations, we believe that the obtained results provide relevant information concerning the role of the microenvironment for renal tumor progression and development.

## MATERIALS AND METHODS

### Reagents

Reagents were from Sigma Chemical Co (St. Louis, MO, USA), tissue culture flasks, dishes, and multi-well plates were from Falcon Orange Scientific (Graignette Business Park, Belgium), and culture media from both tissue and cell lines and supplements were from Gibco BRL (Carlsbad, CA, USA).

### Sample collection and handling

Human adipose tissue explants from cancerous (hRAT; *n* = 14) kidneys were obtained from patients to whom a partial or total (tumor) nephrectomy was performed. Human adipose tissue explants from normal kidneys (hRAN; *n* = 13), were obtained from live kidney donors who had not received previous chemotherapy or radiotherapy treatment. The median of body mass index (BMI) of patients was: 24.2 kg/m^2^ for patients with renal tumor (hRAT), and 26.9 kg/m^2^ for living kidney donors (hRAN). (BMI) (kg/m2) was calculated as weight (kg) divided by height (m) squared.

Samples were transported in PBS with gentamicin (50 μg/ml) and processed immediately. On average, 2 h elapsed from the acquisition of the surgical sample until it was processed under a sterile laminar flow hood. The project was approved by the Medical School’s ethics committee (Universidad Nacional de Cuyo, Argentina). All patients gave their informed consent to undergo tissue harvesting for this research.

### Gene expression by RT-qPCR analysis

Total RNA was extracted from 100 mg of tissue using Trizol reagent (Invitrogen, Carlsbad, CA, USA) and quantified according to its absorbance at 260nm (NanoDrop 2000, Thermo Scientific, Wilmington, USA). Contaminating genomic DNA was degraded with DNAse RQ1 (Promega, Madison, USA), cDNA was synthesized from one microgram of total RNA using 300 pmol oligo-dT primers, 10 mM dNTP (Thermo Scientific, Wilmington, USA) and 200 U M-MLV reverse transcriptase (Promega, Madison, USA). Real-time PCR was performed in a final volume of 20 uL containing 50 ng cDNA, 3mM MgCl2, PCR LightCycler-DNA Master SYBRGreen reaction mix (Roche, Indianapolis, USA) and 0.5 mM of each specific primers (Table 1). Amplification was performed in a using LightCycler thermocycler (Roche, Indianapolis, USA). Controls without reverse transcription were included to ensure that amplifications were from mRNA and not from genomic DNA. Amplicons were characterized according to their melting temperature and size. The mRNA level of each target gene was calculated using the 2ΔΔCt method and normalized against the mRNA of β-actin.

**Table 1:** primer pair sequence are shown for the Forward (F) and Reverse (R) primers used to measure mRNA abundance by RT-qPCR

Gen	Forward (5′-3′)	Reverse (5′-3′)	Ct	Size (pb)	TM (°C)	Gene bank
adiponectin	TGACTCCACCTTCAGAGGCTTT	ATTGACTTTGGGGCTGTTTGGC	35	95	84	NM_004797.2
Leptin	TCACCAGGATCAATGACATTTCAC	CCCAGGAATGAAGTCCAAACC	35	80	82	NM_000230.2
GADPH	GGAGCGAGATCCCTCCAAAAT	GGCTGTTGTCATACTTCTCATGG	35	197	89	NM_002046.3
Actin	CTGTGGCATCCACGAAACTA	AGAAAGGGTGTAACGCAACTA	35	350	89	NM_001101.3
Versican	TGAAGACACACAAGACACGG	CAACGTCCAAACAAGCCTTC	40	119	86	NM_001126336.2

### Immunohistochemistry

10 μm serial cuts were performed on the same tissue samples embedded in paraffin used for H&E staining. Versican, adiponectin, and leptin expression were studied by means of immunohistochemistry. Briefly, hRAN and hRAT microtome slides were first deparaffinized, and then a heat-mediated antigen retrieval, endogenous peroxidase blocking and nonspecific tissue blocking were performed. Slides were then incubated with the different primary antibodies at 4° C, and after that with an anti-rabbit biotinylated secondary IgG antibody. Finally, slides were incubated with peroxidase-conjugated streptavidin. Peroxidase reaction was performed with chromogen 3,3′-diaminobenzidine (DAB) (DAKO LSAB + Kit, HRP). Hematoxylin counter stain was performed. Serial cuts incubated in the absence of primary antibody were used as negative controls. Images were taken with a Nikon Eclipse E200 Microscope fitted with a Micrometric SE Premium (Nikon Corp., Japan) digital still camera at 100× and 400× magnification. DAB staining quantification in the three tissue types was performed in 8–10 fields of each preparation as mentioned above.

### Preparation of conditioned media (CMs) from hRAN and hRAT

Adipose tissues were washed three times with cold PBS to remove red blood cells and debris, and weighed. hRAN or hRAT were plated in culture flask with M199 culture medium (Invitrogen™; 1 g tissue/10 ml M199), supplemented with gentamicin (50 μg/ml) and incubated for 1 h at 37° C in 5% CO_2_. After that, the medium was removed and replaced with fresh medium and the tissues were incubated for 24 h. Subsequently, the supernatants were collected and cells were removed by centrifugation (3 min at 400 × g). Then, supernatants were aliquoted into 1-ml fractions and immediately stored at -80° C. The control-CMs were obtained from the collection of serum-free M199 medium after 24 h of incubation in a culture flask at 37° C in 5% CO_2_.

### Treatment with hRAN- and hRAT-CMs

In order to study cell proliferation, migration and proteins expression of tumor (786-O, ACHN and Caki-1) and non-tumor (HK-2) human renal epithelial cell lines, MCs collected were diluted 1:1 in DMEM-F12 (Invitrogen, UK) 2% fetal bovine serum (FBS; 1% FBS final concentration) and the cells were incubated with the diluted CMs. The experiments were performed with equal volumes of hRAN- and hRAT-CMs. The concentration of total protein in those volumes was quantified using Bradford reagent.

### Culture of tumor and non-tumor renal epithelial cell lines

Tumor (786-O, ACHN and Caki-1) and non-tumor (HK-2) human renal epithelial immortalized cell lines were used. 786-O (ATCC^®^ CRL1932™), ACHN (ATCC^®^ CRL1611™), Caki-1

(ATCC^®^ HTB46™) and HK-2 (ATCC^®^ CRL2190™) were obtained from the American Type Culture Collection (ATCC, Rockville, MD). 786-O is a line derived from a primary clear cell adenocarcinoma (primary tumor); and both ACHN and Caki-1 are lines derived from metastatic sites (pleural effusion and skin respectively. The four cell lines were cultured in DMEM-F12 medium with 10% FBS and 2 μg/ml insulin; and were maintained at 37° C in 5% CO_2_. The number of generations for HK-2 was 13-16; 8–12 for 786-O, 10-14 for ACHN, and 18-21 for Caki-1.

### Proliferation assay

Tumor (3 × 10^3^ 786-O, ACHN or Caki-1 cells/well) and non-tumor (5 × 10^3^ HK-2 cells/well) human renal epithelial cell lines were incubated on 96-well plates with complete DMEM-F12 for 24 h. Then, the four cell lines were treated with hRAN-, hRAT- or control-CMs. After 24 or 48 h, MTT was added to each well at a final concentration of 1 mg/ml and incubated at 37° C for 2 h. The media/MTT solution was then removed without disturbing the attached cells, and accumulated formazan in cells dissolved in acidified (4% 1N HCl) isopropanol. The absorbance of each sample was determined at 570 nm. Each sample was repeated three times. Results are expressed as percentage of color intensity and normalized to cells grown in control-CMs. In addition, we evaluated cell proliferation by the cell count technique using Tripan blue in order to identify dead cells.

### Cell adhesion assay

Adhesion assays were performed following a protocol previously reported [54]. Briefly, 96-well plates were coated with 100 μl hRAN-, hRAT- or control-CMs at 37° C overnight in 5% CO_2_. These CMs were set in three wells and each experiment was repeated three times. Plates were then blocked with 1 mg/ml bovine serum albumin at 37° C for 1 h. After washing with PBS, 786-O, ACHN, Caki-1 and HK-2 cells (5 × 10^4^ cells/well) were suspended in serum-free DMEM-F12 medium, seeded and allowed to adhere to the CMs factors-coated wells at 37° C for 1 h in 5% CO_2_. Non-adherent cells were aspirated and wells washed twice with PBS. Residual cells were evaluated by MTT assay. Cell adhesion to hRAN-, hRAT-CMs factors was expressed as percentage of control-CMs.

### Cell migration assay

The effect of hRAN-, hRAT- or control-CMs on the motility of tumor and non-tumor human renal epithelial cell lines was evaluated by wound-healing and by Transwells migration assays. *Wound-healing assays:* 786-O, ACHN, Caki-1 and HK-2 cells were grown on 96-well plates with complete DMEM-F12. Confluent cell monolayers were wounded with a pipette tip, washed twice with PBS and hRAN-, hRAT- or control-CMs were added. Images at time zero (0 hours) were captured to record the initial width of the wounds. The recovery of the wounded monolayers due to cell migration toward the denuded area was evaluated after 6, 12 and 24 h. The images were acquired by an inverted phase-contrast microscope (Olympus CKX-41) using a 4× objective. Quantification was performed using ImageJ (NIH, Bethesda, MD, USA) by a polygon selection mode. The percentage of the wounded area was determined at 6 or 12 hs respect to control (0 h). *Transwell migration assays:* 786-O, ACHN, Caki-1 and HK-2 cells (2–9 10^5^ cells/0.2 ml) were placed into the top transwell with 8 μm pore membranes (NUNC cat.#140629). They were then incubated with hRAN-, hRAT- or control-CMs and allowed to transmigrate across the porous membrane for 20 h. At the end of the assay, inserts were removed and the cells were fixed in 4% paraformaldehyde and then stained with a 0.1% crystal-violet solution. Tumor and non-tumor cells on the upper membrane surface were removed with a paper towel. The air-dried membranes were viewed under 20× magnification and migrated cells were counted in 5 randomly chosen fields per membrane.

### Preparation of cell lysates from renal epithelial cells after incubation with hRAN-, hRAT- or control-CMs

786-O, ACHN, Caki-1 and HK-2 cells were seeded in six-well plates in DMEM-F12 complete medium. When cells reached 75–80% confluence, the medium was aspirated and cells were washed twice with PBS. Then, cells were incubated at 37° C for 24 h either with hRAN-, hRAT- or control-CMs (50% CM, 50% DMEM-F12 2% FSB). Cells were lysed with Ripa buffer, pelleted by centrifugation at 4° C and stored at −80° C.

### Western blot analysis

#### Protein expression in epithelial cell lines

In order to evaluate protein expression levels, Western blots were performed. AdipoR1, ObR, CD44, vimentin, ERK, pERK, PI3K and pPI3K were measured after incubation of the epithelial cell lines with the different CMs obtained. In order to lyse cells, Ripa buffer was used (Tris 10 mM pH 7,5; NaCl 150 mM; sodium vanadate 2 mM; sodium deoxycholate; SDS 0,1%; igepal 1%; protease inhibitors). Total protein in samples was quantified by Bradford method. Proteins were separated in a SDS-PAGE 12 or 18% gel, and electrotransferred to a nitrocellulose membrane (Amersham). The membrane was later blocked with bovine serum albumin (Sigma-Aldrich, 0055K) and then incubated with the different antibodies ON at 4° C. The membranes were later washed, and incubated with proper secondary antibodies conjugated to HRP. Antibody complexes were visualized by means of chemiluminescence (ECL; GE Helathcare). Bands were quantified by densitometry using FIJI Image processing package [24]. In the cell extracts, β-actin level in samples was used to determine that equal quantities of proteins were loaded in the gel.

### Protein expression in CMs

In addition, adiponectin, ADAMTS1 and perilipin 1 expression was measured in hRAN- and hRAT-CMs. Total protein in samples was quantified by Bradford method. Proteins were separated in a SDS-PAGE 12% gel, and electrotransferred, afterwards we performed the same protocol described for cells. The western blot assay for the hRAN- and hRAT-CMs was performed by loading equal volumes of each CM (40 μl).

### Statistical methods

The statistical significance between different experimental conditions was evaluated by *t-*test or one-way ANOVA. Tukey´s post-hoc tests were performed within each individual treatment. The results are presented as mean ± SEM. Results were considered significant at *p* < 0.05.

## References

[1] Wu Y, Kim JY, Zhou S, Smas CM Differential screening identifies transcripts with depot-dependent expression in white adipose tissues. BMC Genomics. 2008; 9:397. 10.1186/1471-2164-9-397. 18721461 PMC2547859

[2] Park J, Euhus DM, Scherer PE Paracrine and endocrine effects of adipose tissue on cancer development and progression. Endocr Rev. 2011; 32:550–70. 10.1210/er.2010-0030. 21642230 PMC3369575

[3] Kershaw EE, Flier JS Adipose tissue as an endocrine organ. J Clin Endocrinol Metab. 2004; 89:2548–56. 10.1210/jc.2004-0395. 15181022

[4] Finley DS, Calvert VS, Inokuchi J, Lau A, Narula N, Petricoin EF, Zaldivar F, Santos R, Tyson DR, Ornstein DK Periprostatic adipose tissue as a modulator of prostate cancer aggressiveness. J Urol. 2009; 182:1621–27. 10.1016/j.juro.2009.06.015. 19683746

[5] Ibrahim MM Subcutaneous and visceral adipose tissue: structural and functional differences. Obes Rev. 2010; 11:11–18. 10.1111/j.1467-789X.2009.00623.x. 19656312

[6] Schäffler A, Schölmerich J, Buechler C Mechanisms of disease: adipokines and breast cancer - endocrine and paracrine mechanisms that connect adiposity and breast cancer. Nat Clin Pract Endocrinol Metab. 2007; 3:345–54. 10.1038/ncpendmet0456. 17377617

[7] Creydt VP, Sacca PA, Tesone AJ, Vidal L, Calvo JC Adipocyte differentiation influences the proliferation and migration of normal and tumoral breast epithelial cells. Mol Med Rep. 2010; 3:433–39. 10.3892/mmr_00000276. 21472258

[8] Wang YY, Lehuédé C, Laurent V, Dirat B, Dauvillier S, Bochet L, Le Gonidec S, Escourrou G, Valet P, Muller C Adipose tissue and breast epithelial cells: a dangerous dynamic duo in breast cancer. Cancer Lett. 2012; 324:142–51. 10.1016/j.canlet.2012.05.019. 22643115

[9] Pistone Creydt V, Fletcher SJ, Giudice J, Bruzzone A, Chasseing NA, Gonzalez EG, Sacca PA, Calvo JC Human adipose tissue from normal and tumoral breast regulates the behavior of mammary epithelial cells. Clin Transl Oncol. 2013; 15:124–31. 10.1007/s12094-012-0896-x. 22855180

[10] Fletcher SJ, Sacca PA, Pistone-Creydt M, Coló FA, Serra MF, Santino FE, Sasso CV, Lopez-Fontana CM, Carón RW, Calvo JC, Pistone-Creydt V Human breast adipose tissue: characterization of factors that change during tumor progression in human breast cancer. J Exp Clin Cancer Res. 2017; 36:26. 10.1186/s13046-017-0494-4. 28173833 PMC5297209

[11] Campo-Verde-Arbocco F, López-Laur JD, Romeo LR, Giorlando N, Bruna FA, Contador DE, López-Fontana G, Santiano FE, Sasso CV, Zyla LE, López-Fontana CM, Calvo JC, Carón RW, Pistone Creydt V Human renal adipose tissue induces the invasion and progression of renal cell carcinoma. Oncotarget. 2017; 8:94223–34. 10.18632/oncotarget.21666. 29212223 PMC5706869

[12] Fletcher SJ, Hapon MB, Callegari EA, Crosbie ML, Santiso N, Ursino A, Amato AR, Gutiérrez A, Sacca PA, Dreszman R, Pérez A, Carón RW, Calvo JC, Pistone-Creydt V Comparative proteomics of soluble factors secreted by human breast adipose tissue from tumor and normal breast. Oncotarget. 2018; 9:31007–17. 10.18632/oncotarget.25749. 30123423 PMC6089553

[13] Shen XD, Zhang L, Che H, Zhang YY, Yang C, Zhou J, Liang CZ Circulating levels of adipocytokine omentin-1 in patients with renal cell cancer. Cytokine. 2016; 77:50–55. 10.1016/j.cyto.2015.09.004. 26539805

[14] Eheman C, Henley SJ, Ballard-Barbash R, Jacobs EJ, Schymura MJ, Noone AM, Pan L, Anderson RN, Fulton JE, Kohler BA, Jemal A, Ward E, Plescia M, et al Annual Report to the Nation on the status of cancer, 1975-2008, featuring cancers associated with excess weight and lack of sufficient physical activity. Cancer. 2012; 118:2338–66. 10.1002/cncr.27514. 22460733 PMC4586174

[15] Scelo G, Larose TL Epidemiology and Risk Factors for Kidney Cancer. J Clin Oncol. 2018. 10.1200/JCO.2018.79.1905. 30372394 PMC6299342

[16] Greenberg AS, Obin MS Obesity and the role of adipose tissue in inflammation and metabolism. Am J Clin Nutr. 2006; 83:461S–65S. 10.1093/ajcn/83.2.461S. 16470013

[17] Surmacz E Leptin and adiponectin: emerging therapeutic targets in breast cancer. J Mammary Gland Biol Neoplasia. 2013; 18:321–32. 10.1007/s10911-013-9302-8. 24136336

[18] Andò S, Barone I, Giordano C, Bonofiglio D, Catalano S The Multifaceted Mechanism of Leptin Signaling within Tumor Microenvironment in Driving Breast Cancer Growth and Progression. Front Oncol. 2014; 4:340. 10.3389/fonc.2014.00340. 25505738 PMC4245002

[19] Mauro L, Catalano S, Bossi G, Pellegrino M, Barone I, Morales S, Giordano C, Bartella V, Casaburi I, Andò S Evidences that leptin up-regulates E-cadherin expression in breast cancer: effects on tumor growth and progression. Cancer Res. 2007; 67:3412–21. 10.1158/0008-5472.CAN-06-2890. 17409452

[20] Gonzalez-Perez RR, Lanier V, Newman G Leptin’s Pro-Angiogenic Signature in Breast Cancer. Cancers (Basel). 2013; 5:1140–62. 10.3390/cancers5031140. 24202338 PMC3795383

[21] Chung SJ, Nagaraju GP, Nagalingam A, Muniraj N, Kuppusamy P, Walker A, Woo J, Győrffy B, Gabrielson E, Saxena NK, Sharma D ADIPOQ/adiponectin induces cytotoxic autophagy in breast cancer cells through STK11/LKB1-mediated activation of the AMPK-ULK1 axis. Autophagy. 2017; 13:1386–403. 10.1080/15548627.2017.1332565. 28696138 PMC5584870

[22] Palanisamy K, Nareshkumar RN, Sivagurunathan S, Raman R, Sulochana KN, Chidambaram S Anti-angiogenic effect of adiponectin in human primary microvascular and macrovascular endothelial cells. Microvasc Res. 2019; 122:136–45. 10.1016/j.mvr.2018.08.002. 30144414

[23] Catalán V, Gómez-Ambrosi J, Rodríguez A, Frühbeck G Adipose tissue immunity and cancer. Front Physiol. 2013; 4:275. 10.3389/fphys.2013.00275. 24106481 PMC3788329

[24] Gati A, Kouidhi S, Marrakchi R, El Gaaied A, Kourda N, Derouiche A, Chebil M, Caignard A, Perier A Obesity and renal cancer: Role of adipokines in the tumor-immune system conflict. Oncoimmunology. 2014; 3:e27810. 10.4161/onci.27810. 24804162 PMC4010540

[25] McRae N, Forgan L, McNeill B, Addinsall A, McCulloch D, Van der Poel C, Stupka N Glucocorticoids improve myogenic differentiation *in vitro* by suppressing the synthesis of versican, a transitional matrix protein overexpressed in dystrophic skeletal muscles. Int J Mol Sci. 2017; 18:E2629. 10.3390/ijms18122629. 29211034 PMC5751232

[26] Wight TN, Frevert CW, Debley JS, Reeves SR, Parks WC, Ziegler SF Interplay of extracellular matrix and leukocytes in lung inflammation. Cell Immunol. 2017; 312:1–14. 10.1016/j.cellimm.2016.12.003. 28077237 PMC5290208

[27] Touab M, Villena J, Barranco C, Arumí-Uría M, Bassols A Versican is differentially expressed in human melanoma and may play a role in tumor development. Am J Pathol. 2002; 160:549–57. 10.1016/S0002-9440(10)64874-2. 11839575 PMC1850640

[28] Zhang Z, Zhang J, Miao L, Liu K, Yang S, Pan C, Jiao B Interleukin-11 promotes the progress of gastric carcinoma via abnormally expressed versican. Int J Biol Sci. 2012; 8:383–93. 10.7150/ijbs.3579. 22393310 PMC3291855

[29] Li D, Wang X, Wu JL, Quan WQ, Ma L, Yang F, Wu KY, Wan HY Tumor-produced versican V1 enhances hCAP18/LL-37 expression in macrophages through activation of TLR2 and vitamin D3 signaling to promote ovarian cancer progression *in vitro* . PLoS One. 2013; 8:e56616. 10.1371/journal.pone.0056616. 23424670 PMC3570526

[30] Wu YJ, La Pierre DP, Wu J, Yee AJ, Yang BB The interaction of versican with its binding partners. Cell Res. 2005; 15:483–94. 10.1038/sj.cr.7290318. 16045811

[31] Ween MP, Oehler MK, Ricciardelli C Role of versican, hyaluronan and CD44 in ovarian cancer metastasis. Int J Mol Sci. 2011; 12:1009–29. 10.3390/ijms12021009. 21541039 PMC3083686

[32] Hernández D, Miquel-Serra L, Docampo MJ, Marco-Ramell A, Bassols A Role of versican V0/V1 and CD44 in the regulation of human melanoma cell behavior. Int J Mol Med. 2011; 27:269–75. 10.3892/ijmm.2010.577. 21136024

[33] Ricciardelli C, Frewin KM, Tan IA, Williams ED, Opeskin K, Pritchard MA, Ingman WV, Russell DL The ADAMTS1 protease gene is required for mammary tumor growth and metastasis. Am J Pathol. 2011; 179:3075–85. 10.1016/j.ajpath.2011.08.021. 22001177 PMC3260838

[34] Zi X, Lusch A, Blair CA, Okhunov Z, Yokoyama NN, Liu S, Baker M, Huynh V, Landman J Effect of perineoplasm perinephric adipose tissues on migration of clear cell renal cell carcinoma cells: a potential role of WNT signaling. Oncotarget. 2016; 7:53277–88. 10.18632/oncotarget.10467. 27409168 PMC5288185

[35] Sacca PA, Creydt VP, Choi H, Mazza ON, Fletcher SJ, Vallone VB, Scorticati C, Chasseing NA, Calvo JC Human periprostatic adipose tissue: its influence on prostate cancer cells. Cell Physiol Biochem. 2012; 30:113–22. 10.1159/000339051. 22759960

[36] Calle EE, Kaaks R Overweight, obesity and cancer: epidemiological evidence and proposed mechanisms. Nat Rev Cancer. 2004; 4:579–91. Review 10.1038/nrc1408. 15286738

[37] Ouchi N, Parker JL, Lugus JJ, Walsh K Adipokines in inflammation and metabolic disease. Nat Rev Immunol. 2011; 11:85–97. 10.1038/nri2921. 21252989 PMC3518031

[38] Obeid S, Hebbard L Role of adiponectin and its receptors in cancer. Cancer Biol Med. 2012; 9:213–20. 10.7497/j.issn.2095-3941.2012.04.001. 23691481 PMC3643674

[39] Polyakova E, Belyaeva O, Bazhenova E, Berkovich O, Berezina A, Ionin V, Bakulina A, Baranova E, Shlyakhto E Insulin resistance and low serum adiponectin level as a risk factors of atherosclerosis and metabolic syndrome. Atherosclerosis. 2017; 263:e252–53. 10.1016/j.atherosclerosis.2017.06.820.

[40] Katira A, Tan PH Adiponectin and its receptor signaling: an anti-cancer therapeutic target and its implications for anti-tumor immunity. Expert Opin Ther Targets. 2015; 19:1105–25. 10.1517/14728222.2015.1035710. 25952656

[41] Hancke K, Grubeck D, Hauser N, Kreienberg R, Weiss JM Adipocyte fatty acid-binding protein as a novel prognostic factor in obese breast cancer patients. Breast Cancer Res Treat. 2010; 119:367–7. 10.1007/s10549-009-0577-9. 19842034

[42] Basen-Engquist K, Chang M Obesity and cancer risk: recent review and evidence. Curr Oncol Rep. 2011; 13:71–76. 10.1007/s11912-010-0139-7. 21080117 PMC3786180

[43] Horiguchi A, Sumitomo M, Asakuma J, Asano T, Zheng R, Asano T, Nanus DM, Hayakawa M Leptin promotes invasiveness of murine renal cancer cells via extracellular signal-regulated kinases and rho dependent pathway. J Urol. 2006; 176:1636–41. 10.1016/j.juro.2006.06.040. 16952706

[44] Matsunaga H, Iwashita M, Shinjo T, Yamashita A, Tsuruta M, Nagasaka S, Taniguchi A, Fukushima M, Watanabe N, Nishimura F Adipose tissue complement factor B promotes adipocyte maturation. Biochem Biophys Res Commun. 2018; 495:740–48. 10.1016/j.bbrc.2017.11.069. 29137982

[45] Fujisaki K, Fujimoto H, Sangai T, Nagashima T, Sakakibara M, Shiina N, Kuroda M, Aoyagi Y, Miyazaki M Cancer-mediated adipose reversion promotes cancer cell migration via IL-6 and MCP-1. Breast Cancer Res Treat. 2015; 150:255–63. 10.1007/s10549-015-3318-2. 25721605

[46] Zizola CF, Julianelli V, Bertolesi G, Yanagishita M, Calvo JC Role of versican and hyaluronan in the differentiation of 3T3-L1 cells into preadipocytes and mature adipocytes. Matrix Biol. 2007; 26:419–30. 10.1016/j.matbio.2007.04.002. 17513099

[47] Musil KJ, Malmström A, Donnér J Alteration of proteoglycan metabolism during the differentiation of 3T3-L1 fibroblasts into adipocytes. J Cell Biol. 1991; 114:821–26. 10.1083/jcb.114.4.821. 1714464 PMC2289887

[48] Iida J, Clancy R, Dorchak J, Somiari RI, Somiari S, Cutler ML, Mural RJ, Shriver CD DNA aptamers against exon v10 of CD44 inhibit breast cancer cell migration. PLoS One. 2014; 9:e88712. 10.1371/journal.pone.0088712. 24586375 PMC3929491

[49] Nam K, Oh S, Lee KM, Yoo SA, Shin I CD44 regulates cell proliferation, migration, and invasion via modulation of c-Src transcription in human breast cancer cells. Cell Signal. 2015; 27:1882–94. 10.1016/j.cellsig.2015.05.002. 25979842

[50] Matsumoto Y, Itou J, Sato F, Toi M SALL4 - KHDRBS3 network enhances stemness by modulating CD44 splicing in basal-like breast cancer. Cancer Med. 2018; 7:454–62. 10.1002/cam4.1296. 29356399 PMC5806117

[51] Gravina GL, Mancini A, Ranieri G, Di Pasquale B, Marampon F, Di Clemente L, Ricevuto E, Festuccia C Phenotypic characterization of human prostatic stromal cells in primary cultures derived from human tissue samples. Int J Oncol. 2013; 42:2116–22. 10.3892/ijo.2013.1892. 23589051

[52] Satelli A, Li S Vimentin in cancer and its potential as a molecular target for cancer therapy. Cell Mol Life Sci. 2011; 68:3033–46. 10.1007/s00018-011-0735-1. 21637948 PMC3162105

[53] Thiery JP Epithelial-mesenchymal transitions in tumour progression. Nat Rev Cancer. 2002; 2:442–54. 10.1038/nrc822. 12189386

[54] Retta SF, Ternullo M, Tarone G Adhesion to matrix proteins. Methods Mol Biol. 1999; 96:125–30. 10.1385/1-59259-258-9:125. 10098129

